# Baculovirus-Induced Climbing Behavior Favors Intraspecific Necrophagy and Efficient Disease Transmission in *Spodoptera exigua*

**DOI:** 10.1371/journal.pone.0136742

**Published:** 2015-09-24

**Authors:** Dulce Rebolledo, Rodrigo Lasa, Roger Guevara, Rosa Murillo, Trevor Williams

**Affiliations:** 1 Instituto de Ecología AC, Xalapa, Veracruz, 91070, Mexico; 2 Instituto de Agrobiotecnología (CSIC), Avda. de Pamplona, Mutilva, 31192, Pamplona, Spain; Institut National de la Recherche Agronomique (INRA), FRANCE

## Abstract

Shortly prior to death, many species of Lepidoptera infected with nucleopolyhedrovirus climb upwards on the host plant. This results in improved dissemination of viral occlusion bodies over plant foliage and an increased probability of transmission to healthy conspecific larvae. Following applications of *Spodoptera exigua multiple nucleopolyhedrovirus* for control of *Spodoptera exigua* on greenhouse-grown sweet pepper crops, necrophagy was observed by healthy *S*. *exigua* larvae that fed on virus-killed conspecifics. We examined whether this risky behavior was induced by olfactory or phagostimulant compounds associated with infected cadavers. Laboratory choice tests and olfactometer studies, involving infected and non-infected cadavers placed on spinach leaf discs, revealed no evidence for greater attraction of healthy larvae to virus-killed over non-infected cadavers. Physical contact or feeding on infected cadavers resulted in a very high incidence of transmission (82–93% lethal disease). Observations on the behavior of *S*. *exigua* larvae on pepper plants revealed that infected insects died on the uppermost 10% of foliage and closer to the plant stem than healthy conspecifics of the same stage, which we considered clear evidence of baculovirus-induced climbing behavior. Healthy larvae that subsequently foraged on the plant were more frequently observed closer to the infected than the non-infected cadaver. Healthy larvae also encountered and fed on infected cadavers significantly more frequently and more rapidly than larvae that fed on non-infected cadavers. Intraspecific necrophagy on infected cadavers invariably resulted in virus transmission and death of the necrophagous insect. We conclude that, in addition to improving the dissemination of virus particles over plant foliage, baculovirus-induced climbing behavior increases the incidence of intraspecific necrophagy in *S*. *exigua*, which is the most efficient mechanism of transmission of this lethal pathogen.

## Introduction

Baculoviruses are dsDNA viruses that infect insects, particularly the larvae of butterflies and moths (Lepidoptera) [1]. Larvae usually become infected by feeding on foliage contaminated with virus occlusion bodies (OBs). During the infection process larvae change color, development and feeding activity slows and mobility is reduced [2]. Shortly prior to death, infected larvae migrate to the top of the plant, where they die in a characteristic form hanging from the pseudopods, a behavior induced by the baculovirus [3]. Recently, a virus gene (*egt*) was identified as being responsible for this behavior in Gypsy moth larvae, *Lymantria dispar* L. [4], but not in other species of Lepidoptera infected by *Autographa californica multiple nucleopolyhedrovirus* [5, 6]. Increased locomotor behavior prior to death is a similar but distinct baculovirus-induced behavior, that is modulated by another virus gene (*ptp*) in *Spodoptera exigua* and *Bombyx mori* [7–9].

Following death, viral OBs are released into the environment and fall, or are washed by rainfall, on to the leaves of the lower parts of the plant, where the infection can be transmitted horizontally to susceptible conspecifics [10]. Other routes of horizontal transmission of these viruses include the consumption of plant material contaminated by the feces or regurgitate of virus-infected larvae [11], or through interactions with insect natural enemies [12, 13].

Certain species of Lepidoptera show cannibalistic behavior, particularly during the final larval stages, or in situations of low food availability or high population density [14, 15]. The ecological and evolutionary consequences of this behavior have been examined with reference to the effects of diet [16], host plant [17], secondary plant chemistry [18, 19], the presence of toxins [20], development and molting [21], endocrinological effects [22], the impact of parasitism [23, 24], or the risks of predation [25], among others. Cannibalism also is a route for the transmission for certain pathogens, including baculoviruses, when larvae consume infected conspecifics [26–28].

The general term cannibalism, or intraspecific predation, defined as the process of consuming all, or part of a conspecific [29], is a behavior that may occur in different contexts. For example, in cases in which individuals kill and consume conspecifics, this activity carries risks of personal injury or reduced inclusive fitness for the cannibal that consumes kin [30]. In contrast, the consumption of a conspecific that has died from some other cause can best be termed intraspecific necrophagy [31], and this is the terminology that we adopt in this study.

The *Spodoptera exigua multiple nucleopolyhedrovirus* (SeMNPV) (genus *Alphabaculovirus*, family Baculoviridae), is a species-specific lethal pathogen of the beet armyworm *S*. *exigua* (Lepidoptera: Noctuidae) [32], that is used as a biological insecticide against this pest [33]. Cannibalism has been observed in late instars of *S*. *exigua*, particularly when reared at high densities in the laboratory [22]. The prevalence of cannibalism by this pest in the field is uncertain, but following applications of a SeMNPV-based insecticide in greenhouse grown crops, intraspecific necrophagy of virus-killed insects by healthy conspecifics can be readily observed (R. Lasa, pers. obs.). These observations indicate that virus-killed cadavers may be attractive to *S*. *exigua* larvae, despite the seemingly high risk of disease transmission that they pose to healthy conspecifics.

The aim of this study was to examine intraspecific necrophagy in direct choice experiments and to test for evidence for a volatile attractant or feeding stimulant in virus-killed insects that may promote this behavior. Using simulated greenhouse conditions, we then examined the relationship between pathogen-mediated manipulation of larval climbing behavior (also known as tree-top disease) and pest foraging activity on the frequency of necrophagy and the likelihood of transmission of this pathogen.

## Material and Methods

### Insect colony and virus strain

Larvae were obtained from a laboratory colony of *S*. *exigua*, started in 2012 using larvae originally collected from maize fields close to Monterrey, Mexico. The collection of insects was authorized by the owner of the land and did not involve any endangered or protected species. This colony was reared under controlled conditions of 25 ± 2°C temperature, 70 ± 5% relative humidity and 12:12 light-dark photoperiod, on a semisynthetic diet adapted from Hoffman’s tobacco hornworm diet [33]. Adults were fed *ad libitum* on a 10% (wt/vol) sucrose solution.

SeMNPV OBs were kindly supplied by P. Támez-Guerra (Universidad Autónoma de Nuevo León, Mexico) and used to inoculate fourth instar *S*. *exigua* larvae that were subsequently reared on diet until death. Virus deaths were identified by the characteristic liquefaction of the insect tegument and, when in doubt, were confirmed by direct observation of Giemsa-stained OBs using a phase-contrast microscope [2]. Virus-killed larvae were triturated and OBs were purified as described previously [34]. The OBs were suspended in distilled water, counted using a Neubauer Improved chamber (Hawsksley, Lancing, United Kingdom) and stored at 4°C prior to use. DNA extracted from these OBs and analyzed using the restriction endonucleases *Bgl*II and *Pst*I (described previously [34]) indicated that this strain of SeMNPV was identical in terms of restriction profiles to that of SeMNPV-US2 (data not shown), which is the principal active ingredient of the biological insecticide Spod-X (Certis USA LLC, Columbia, MD). All experiments were performed in the laboratory facilities at the Instituto de Ecología AC, Xalapa, Veracruz, Mexico (19° 30’ 46.3” N, 96° 56’ 34.8” W).

### Production of infected and non-infected insect cadavers

To produce insect cadavers infected with SeMNPV, groups of 50 fourth instars were separated and individually allowed to feed on a slice of diet (10 x 10 x 2 mm) that had been previously contaminated on the upper surface with 10 μl of a suspension of 1x10^8^ OB/ml. After 24 h, a new piece of untreated diet was supplied to the larvae. When larvae died due virus infection, vials were frozen at -20°C to avoid lysis of the insect tegument. Another group of fourth instar larvae (controls) was reared on untreated diet (4 days), until they reached the fifth instar, and were then individualized and stored at -20°C.

### Preference test: Infected vs. non-infected cadavers

To assess whether larvae were attracted to infected or non-infected cadavers, a Petri dish choice test was performed. Spinach plants (*Spinacea oleracea* L.) purchased in a local supermarket were decontaminated in 0.1% (wt/vol) sodium hypochlorite for 5 min and then rinsed under running water for 10 min. Two leaf discs of spinach were cut from leaves that showed the same texture and color, using a 20 mm diameter cork borer. Discs were placed on opposite sides of the Petri dish and one frozen infected cadaver was placed at the center of one disc and a frozen non-infected cadaver was placed at the center of the other disc. Cadavers were allowed to thaw fully at room temperature. A recently molted fourth instar insect from the laboratory colony was then placed in the center of the Petri dish and observed continuously during a 30 min period. Physical contact with the cadaver was recorded, as were acts of necrophagy and the response time from introduction until each type of behavior was observed. At the end of the observation period, each larva was placed individually in a 50 ml plastic container with a small block of diet, reared at 25 ± 2°C until pupation or death due to virus infection. The choice test was performed on 97 occasions. Treatments were switched between each side of the Petri dish on each occasion.

### Feeding stimulant effects

To assess whether virus-infected cadavers contained a feeding stimulant, spinach leaf discs were prepared as described above. Each of four discs was divided into two equal sectors using a non-toxic fine tipped marker. Discs were placed at points 40 mm equidistant from the center of a Petri dish so that the dividing line of each disc was directed towards the center of the dish. One half of each disc was painted, using a small brush, with ~20 μl of a crude suspension of OBs that was obtained directly from a dead infected larva and the other half was covered with distilled water as a control. Treatments were assigned at random to each side of the leaf disc. When treatments had dried, a recently-molted fourth instar larva from the laboratory colony was placed in the center of the Petri dish and allowed to feed for 24 hours at 25 ± 2°C under 960 lux ilumination in an insectary room. The sum of feeding events that the larvae made in each treatment of the four discs was counted. Feeding events were identified by discrete perforations made in the parenchyma of the OB-contaminated and control sides of each leaf disc. The test was performed a total of 45 times.

Another experiment was performed with the same methodology, but in this case, the control side of each leaf disc was painted with ~20 μl of homogenate of non-infected larval cadavers, instead of water. The other half of each disc was covered with a suspension obtained from crude infected cadavers as described above. In this case, in addition to counting the number of feeding events during a 24 h feeding period, the leaf area consumed in each disc treatment was measured using high resolution photos with the ImageJ program [35]. The test was performed a total of 45 times.

### Response to volatile components of infected vs. non-infected cadavers

To determine the attraction of healthy larvae to volatile compounds of cadavers, a Y tube olfactometer was constructed from transparent non-absorbent acrylic with a rectangular cross-section, 20 cm long and 3x3 cm wide, with two arms 10 cm long separated by a 45° internal angle. Incoming air, from a small aquarium pump (Elite 800, Grupo Acuario Lomas, Mexico), was filtered through activated charcoal and split equally between two, 450 ml glass holding chambers (flasks). One chamber containing a single non-infected cadaver on a spinach leaf disc served as a control whereas the other chamber held the test material comprising a single virus-killed cadaver on a spinach leaf disc. The air passed from each chamber into the respective arms of the Y tube, before entering the main tube of the olfactometer. Airflow was maintained at 400 ml/min by two inline flowmeters (Cole Parmer Instrument Co., Chicago, IL).

Holding chambers were closed to concentrate the volatile for five minutes, and then, the airflow was passed through the odor source into the arms of the olfactometer. A recently molted fourth instar larva from the laboratory colony was placed at the base of the main tube and allowed to respond for 15 minutes. A positive response was recorded when a larva crossed a pre-defined choice line located at an arbitrary distance of 7 cm along each of the arms of the Y-tube section of the olfactometer, as is usual for Y tube olfactometer studies with invertebrates [36]. After each replicate the olfactometer was cleaned using neutral detergent and water, and arms rotated 180° to avoid a position effect. A total of 55 replicates were performed.

### Baculovirus-induced climbing and foraging behavior of larvae on plants

This study was conducted to determine the spatial distribution of healthy and infected larvae of *S*. *exigua* on the plant and the relationship of these distributions with the foraging behavior of healthy conspecifics. The experiment was conducted on sweet pepper plants (var. Annum) between 45 and 83 cm height and with an average of 34 true leaves. The plants were grown in a mixture of compost, soil and volcanic pumice (2:1:1) in sturdy plastic bags (20 x 25 cm) placed inside cages of 60 x 60 cm wide and 1 m height and covered with a nylon mesh with a 1 mm pore size. The test was carried out in an environmental simulation laboratory (16 m^2^ area) under controlled conditions, (25 ± 2°C) temperature, (70 ± 5%) relative humidity and 12:12 h light-dark photoperiod. Two fourth instar larvae were placed simultaneously on the stem at the center of each plant; one larva had been infected three days before with a suspension of 2.6x10^7^ OBs/ml using the droplet bioassay technique [37], the other was a healthy larva of the same age. Larvae were observed at 2 h intervals until the infected larvae died and showed no movement. At this moment, the healthy larva was sacrificed by crushing the cephalic capsule with entomological forceps, at the point on the plant where it was observed at the moment of death of the infected conspecific.

The positions of the infected and non-infected larvae on the plant at the moment of death were noted. For this, the vertical distance between the base of the plant and each cadaver was measured as was the horizontal distance between the cadavers and the plant stem.

A recently-molted fourth instar larva was then immediately placed on the stem at the center of the plant and allowed to move freely. The larva was observed at 1 h intervals for a period of 48 h. Hourly nocturnal observations were performed using six 250 W red light bulbs placed at a distance of 2.5 m above the plants. At each observation, the activity of the larva was classified as feeding, walking or resting. An act of necrophagy was recorded when a larva was observed feeding on a cadaver during one or more sequential observations without interruption. In cases in which the larva was observed to cease feeding and move away from the cadaver (>5 mm) during one or more observations, and then return to the cadaver and recommence feeding, each event was considered as a distinct act of necrophagy. Finally, the location of the larva at each observation was recorded in terms of vertical and horizontal distances, as described above. The shortest direct line distance between the healthy larva and each of the cadavers (infected and non-infected) was also noted.

In a few cases in which healthy larvae fell off the plant, they were placed at the base of the stem so that they could climb back onto the plant. After 48 h of hourly observations, larvae were individualized in 50 ml plastic cups with a small block of diet and reared under laboratory conditions until they reached the pupal stage or died due to virus infection. A total of 30 replicates were performed involving a total of 1440 hours of observation.

### Data analysis

The frequency of response of larvae to leaf discs with cadavers was analyzed by χ^2^ test. Mean larval response times to each type of cadaver were compared by t-test. The number of feeding events and the leaf area consumed in each segment of the leaf discs were also compared by t-test. The position of the larvae on the host plant (vertical and horizontal distances, averaged for each replicate) was subjected to analysis of variance (ANOVA). Assumptions of normality and homoscedasticity were examined in all cases. The frequency of observations on which the experimental larva was closer to the location of each type of cadaver (infected and non-infected) was summed for each insect during the experimental period and a generalized linear model was fitted with a binomial error structure specified. As such, each insect was considered to be a single replicate. The significance of changes in model deviance following fitting of explanatory variables were determined with reference to χ^2^ statistics. The mean numbers of acts of necrophagy performed on each type of cadaver were not normally distributed and were compared by Mann-Whitney U-test. Finally, χ^2^ tests were conducted on the prevalence of larvae that performed necrophagous acts and the prevalence of mortality in insects that consumed each type of cadaver.

## Results

### Preference test: Infected vs. non-infected cadavers

**Fig 1:**
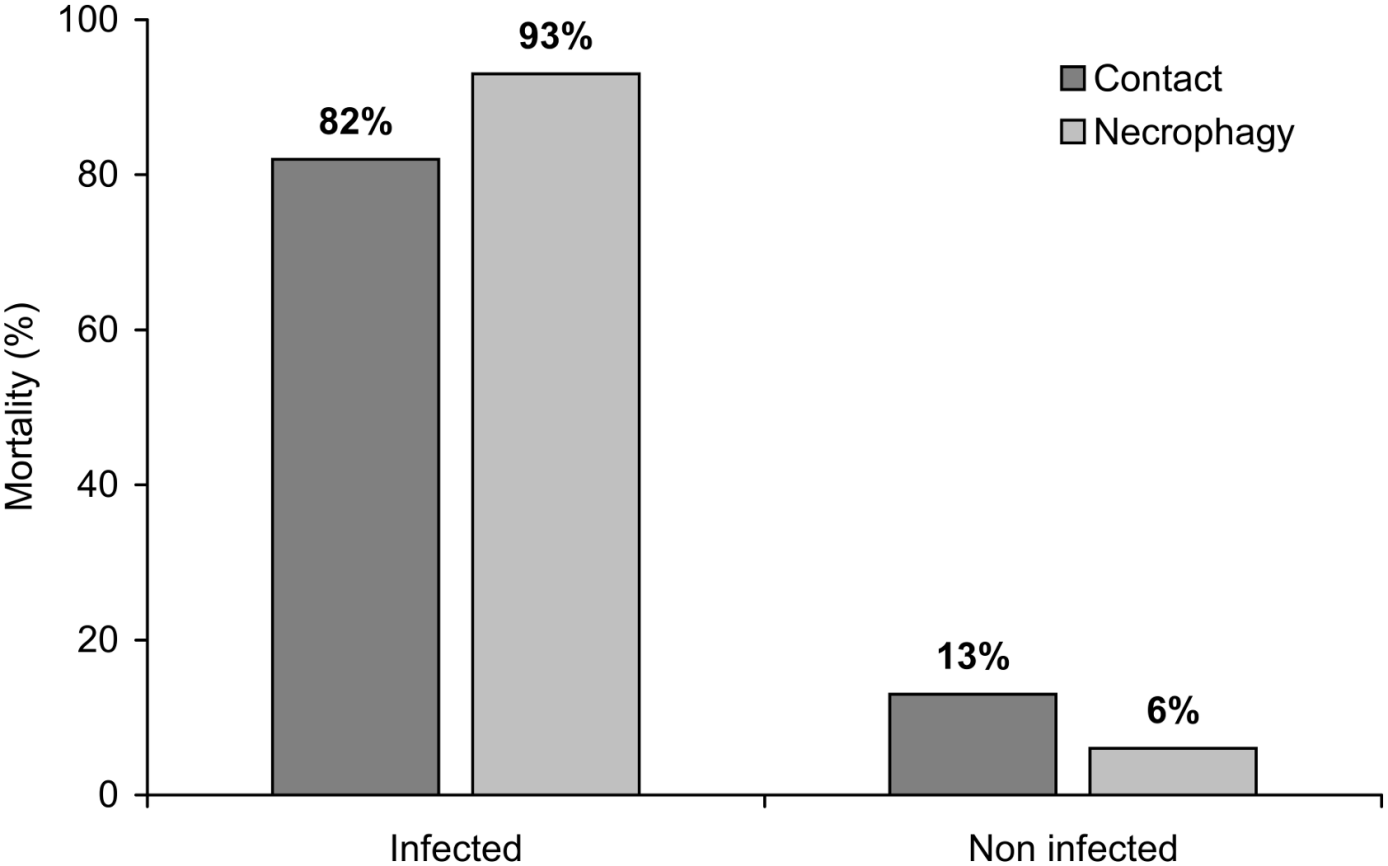
Percentage of mortality of *S*. *exigua* larvae that fed on (necrophagy) or had physical contact with infected and non-infected cadavers (n = 82).

Of the 97 insects tested, 48 (55%) moved towards the leaf disc with the infected cadaver and 39 (45%) selected the disc with a non-infected cadaver (*χ* ^*2*^ = 0.931, *df* = 1, *P* = 0.33), whereas 10 larvae did not respond and were eliminated from the analysis. Of the larvae that selected the infected cadaver, 33 (69%) had physical contact with the cadaver and 15 (31%) were observed to feed on the cadaver. Among larvae that selected the non-infected cadaver, 31 (79%) had physical contact with the cadaver and 8 (21%) fed on the dead insect. The prevalence of feeding on each type of cadaver was similar (*χ* ^*2*^ = 1.275, *df* = 1, *P* = 0.259). The prevalence of virus induced mortality did not differ between larvae that had contact with infected cadavers (82% mortality) and those that fed on infected cadavers (93% mortality) (*χ* ^*2*^ = 0.002, *df* = 1, *P* = 0.96). The larvae that only had contact or fed on the non-infected cadavers subsequently died of virus infection at a prevalence of 13% and 6%, respectively (Fig 1). The mean (± SE) response time to arrive at infected cadavers (579 ± 63 sec) and non-infected cadavers (760 ± 71 sec) was similar (*t* = 1.8, *df* = 80, *P* = 0.07).

### Feeding on virus contaminated vs. non-contaminated foliage

**Fig 2:**
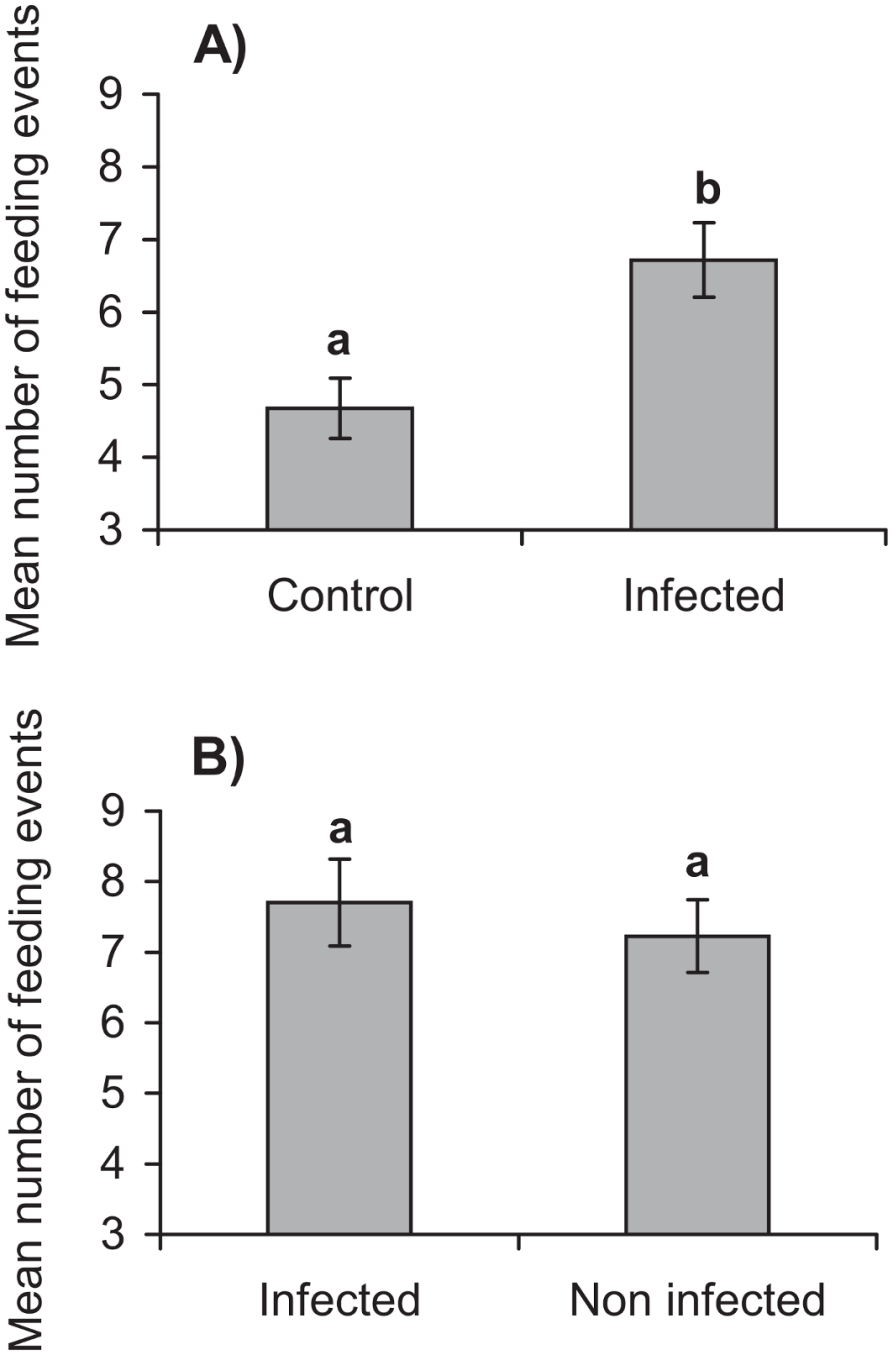
Number of feeding events (+ SE) of *S*. *exigua* larvae on spinach discs with different treatments. a) disc contaminated with virus-infected cadaver vs water (control) (n = 45); b) disc contaminated with infected cadaver vs non-infected cadaver (n = 45). Different letters indicate significant differences between groups (t test, p <0.05).

One larva did not feed on either leaf disc and was eliminated from the analysis. A greater number of feeding events were observed in the segment contaminated with a crude preparation of virus-killed insects than on segments treated with water (*t* = 3.13, *df* = 88, *P* = 0.002) (Fig 2A). However, in a subsequent experiment, no significant differences (*t* = 0.52, *df* = 88, *P* = 0.433) were observed in the mean number (± SE) of feeding events in the segment contaminated with virus-killed insect homogenate (7.2 ± 0.5) or homogenate of non-infected insects (7.7 ± 0.6) (Fig 2B). Similarly, no significant differences were detected in the mean (± SE) total surface area consumed by larvae on segments contaminated with virus-killed insect homogenate (32.1 ± 2.0 mm^2^) or homogenate of non-infected insects (30.3 ± 2.2 mm^2^) (*t* = 0.771, *df* = 88, *P* = 0.291).

### Response to volatile components of infected vs. non-infected cadavers

No significant difference was observed in the response of larvae to volatiles produced by spinach discs with infected cadavers (56% positive response) and spinach discs with non-infected cadavers (44%) (*χ* ^*2*^ = 0.890, *df* = 1, *P* = 0.345). Similarly, the mean (± SE) response time to infected cadavers (191 ± 23 seconds) and non-infected cadavers (202 ± 33 seconds) was similar (*t* = 0.263, *df* = 53 *P* = 0.79).

### Baculovirus-induced climbing and foraging behavior of larvae on plants

**Fig 3:**
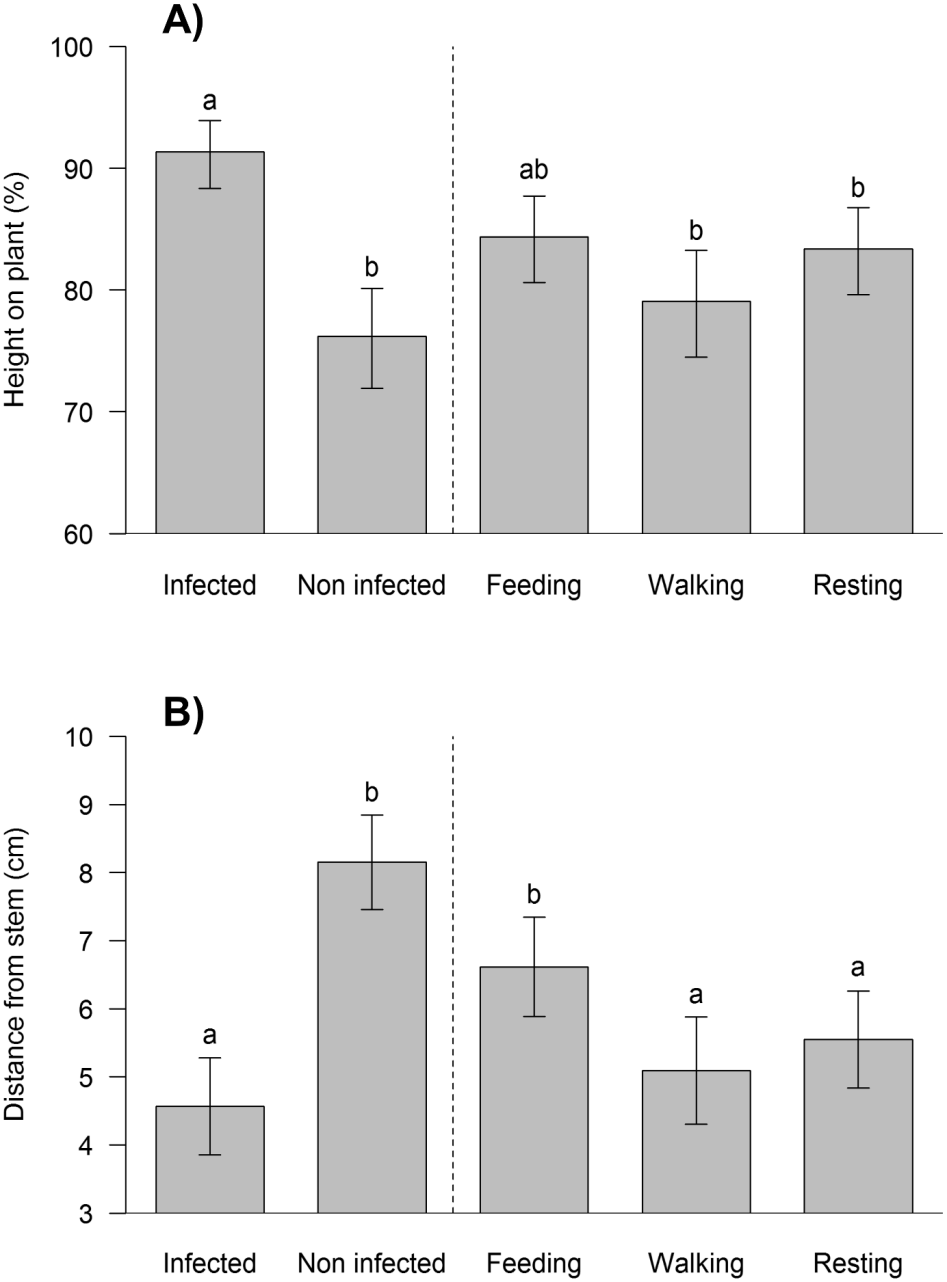
Distribution of live larvae on plant when they were eating, walking and resting, as well as the position of the cadavers (infected and uninfected), (n = 23). a) Height on plant, the scale is presented in terms of relative height with respect to the total height of each plant. b) Distance from stem (cm). Different letters indicate significant differences (ANOVA, p <0.05).

Of the 30 insects (replicates), 8 fell off the plant and did not climb back on during the 48 h observation period; these insects were eliminated from the study, leaving 22 active larvae and a total of 1056 hours of observation. As plants varied in height, the vertical position of each insect was expressed as percentage of total plant height (Fig 3A), with 100% being the topmost point on the plant. The vertical distribution of larvae on the plants varied significantly according to infection status (non-infected vs. infected cadavers) and the activity of the healthy insects (*F* = 3.33, *df* = 4, 95, *P* = 0.01). On average, infected larvae died on the upper 10% of the plant, whereas at the moment of death of the infected insect, non-infected larvae were observed at a significantly lower height, in the upper middle part of the plant (at ∼75% of the total height of the plant), (*t* = 3.2, *df* = 95, *P* = 0.001). At this moment, non-infected larvae were sacrificed *in situ*.

Walking or resting took place at a height that was similar to the height at which the non-infected larvae were sacrificed, whereas feeding tended to occur at sites that were intermediate between the infected and non-infected cadavers and which did not differ significantly from the mean height of either the infected (*t* = 1.83, *df* = 95, *P* = 0.06) or non-infected (*t* = 1.19, *df* = 95, *P* = 0.23) cadavers (Fig 3A).

The horizontal position of larvae also differed according to infection status and the activity of healthy insects (*F* = 3.94, *df* = 4, 95, *P* = 0.005) (Fig 3B). Infected larvae died significantly closer to the stem when compared with the position where non-infected larvae were sacrificed (*t* = 3.53, *df* = 95, *P* < 0.001). The larvae released subsequently, usually fed at sites away from the central stem that did not differ significantly from the average horizontal position of non-infected cadavers (*t* = 1.24, *df* = 95, *P* = 0.21). In contrast, when walking (*t* = 0.41, *df* = 95, *P* = 0.68) or resting (*t* = 0.97, *df* = 95, *P* = 0.33), larvae were closer to the central stem, at a horizontal distance that did not differ significantly from that of infected cadavers.

**Fig 4:**
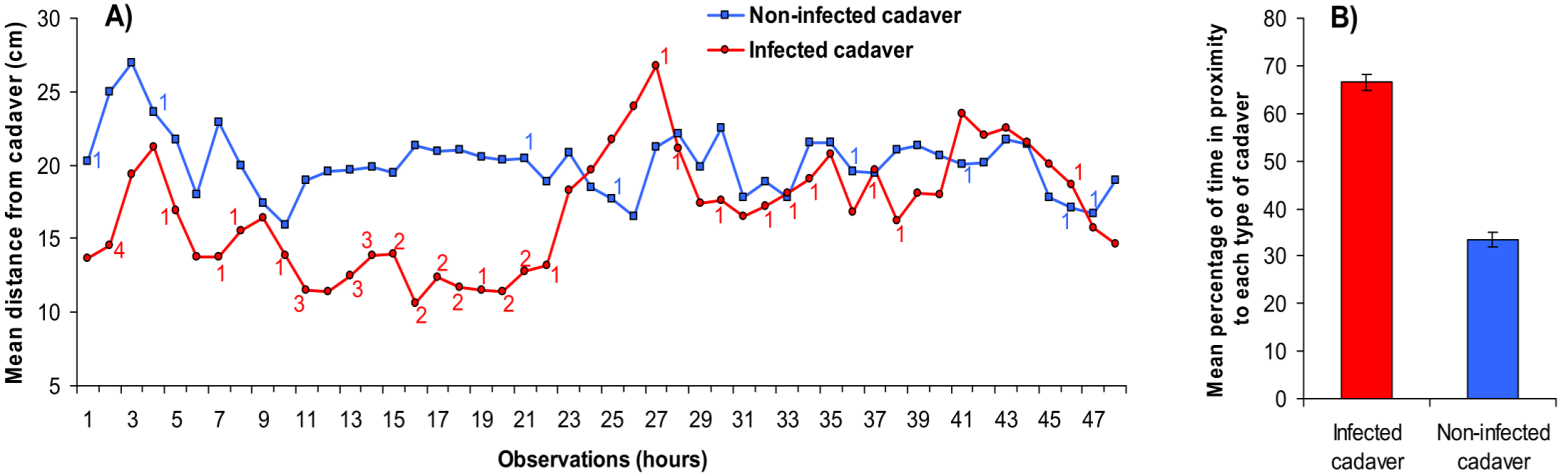
Mean distance between healthy larvae and infected or non-infected cadavers. a) Hourly means of observations performed over a 48 h period on each larva (n = 22 larvae). Points labeled with numerical values indicate number of acts of necrophagy observed at each time point (values shown in blue refer to necrophagy on non-infected cadavers, values in red refer to necrophagy on infected cadavers); b) Mean percentage of observations in which the experimental insect was closer to the infected (red column) or non-infected (blue column) cadaver. Vertical bars indicate SE.

The straight-line distance was measured between healthy larvae and infected and non-infected cadavers at each hourly observation (Fig 4A). The frequencies with which each larva was closer to the infected or non-infected cadaver were compared by fitting a generalized linear model with a binomial error structure specified. On average, larvae were observed to be closer to the infected cadaver on two-thirds (66.5%) of occasions and closer to the non-infected cadaver on one third (33.5%) of occasions, indicating a significant tendency to be closer to the infected cadaver than expected given a random distribution on the plant (*χ* ^*2*^ = 175.1, *df* = 1, *P* < 0.001) (Fig 4B).

Of the 22 larvae that were observed during the experiment, 12 (55%) fed only on the infected cadaver, while 4 larvae (18%) fed only on the non-infected cadaver, 4 larvae (18%) fed on both types of cadaver and 2 larvae did not feed on either cadaver. Overall 48 acts of necrophagy were observed during the 1056 h observation period (Fig 4A). Necrophagous larvae performed an average of 2.3 ± 0.3 acts of necrophagy on the infected cadaver during the observation period, which was significantly more frequently than acts of necrophagy on the non-infected cadaver that were never performed more than once (Mann-Whitney U = 20, P = 0.007), i.e. larvae never returned to the non-infected cadaver to resume necrophagous feeding, whereas 13 out of 16 larvae that fed on the infected cadaver did so on more than one occasion. Necrophagy also occurred significantly more rapidly towards infected cadavers; 13 out of 16 larvae fed on infected cadavers during the first 24 h, compared to 3 out of 8 larvae that fed on non-infected cadavers during the same period (*χ* ^*2*^ = 4.59, *df* = 1, *P* = 0.032). Mortality caused by virus infection was 100% in those larvae that fed on infected cadavers and 50% in those that consumed the non-infected corpse. Of the three larvae that did not practice any act of intraspecific necrophagy, one subsequently died of lethal polyhedrosis disease. As the laboratory insect colony was free of virus disease, observations on virus acquisition by insects that had no direct contact with the infected cadaver were likely a result of contamination of plant surfaces by virus-containing feces and regurgitate of the infected larvae that foraged over the plant prior to death.

## Discussion

Greenhouse observations by one of us had indicated that conspecific necrophagy may be common in *S*. *exigua*, especially following applications of a virus-based insecticide. This behavior appears highly risky given the lethality of the SeMNPV virus. This led us to ask whether virus-killed larvae were particularly attractive to healthy conspecifics which we examined using choice arena and olfactometer tests.

Laboratory studies in Petri dish arenas indicated no differences in the frequencies of selection, contact or necrophagous feeding on infected and non-infected cadavers. However, both physical contact and feeding on infected cadavers resulted in a high prevalence of lethal virus infection in experimental insects. Low levels of virus infection were even observed in insects that had no direct contact with the infected cadaver, indicating that moving around the arena was sufficient for virus transmission in some cases. This reflects the very high pathogenicity of this virus [38].

When offered leaf discs on which one sector had been treated with the remains of infected cadavers, larvae showed a clear preference for the sectors treated with infected cadavers over control sectors, although this preference disappeared when offered a choice between sectors treated with infected and non-infected larvae. This suggests that healthy *S*. *exigua* larvae are attracted to insect remains whether virus-contaminated or not. In a previous study on cannibalism by final instar *S*. *frugiperda* larvae, no evidence was found for discrimination between infected and non-infected conspecifics, both living or dead, whereas an earlier instar avoided necrophagy of virus-killed larvae, suggesting that this response may be stage specific in some species [26].

Similarly, we found no evidence for differential responses to volatiles emitted by infected and non-infected cadavers placed on leaf discs, suggesting that attraction to virus-killed conspecifics is not mediated by one or more pathogen-related volatile compounds. Although insect pathogenic viruses have not been reported to produce volatile compounds that favor their transmission, a number of plant pathogenic viruses release compounds that attract their insect vectors to infected plants [39]. Similarly, studies on insect cadavers infected by the fungal pathogen *Beauveria bassiana* suggests a role of volatile compounds released by the fungus in attracting adult mosquitoes which then become infected [40], whereas termites, flies, and predatory ladybeetles can detect pathogenic fungi by olfaction and strongly avoid contact with contaminated substrates [41–44].

The findings of choice test and olfactometer studies contrasted strongly with observations performed on pepper plants under simulated greenhouse conditions. Studies on plants revealed the importance of the interaction of baculovirus-induced climbing behavior and larval foraging on intraspecific necrophagy in this pest. Encounters between *S*. *exigua* larvae and infected cadavers on pepper plants were invariably followed by intraspecific necrophagy that was shown to be a highly efficient route of transmission of SeMNPV.

Baculovirus-induced climbing behavior resulted in infected insects dying in the upper 10% of the plant, which was significantly higher up the plant than the site at which non-infected conspecifics were located at the moment of death of the diseased insect. This was because non-infected insects were often engaged in walking or resting behaviors at intermediate heights any particular moment, whereas infected larvae tended to be located at higher parts of the plant at death due to the climbing activity elicited by the virus infection. Feeding by healthy larvae generally occurred in the upper 15% section of the plant (Fig 3A) in which young leaves are present. These leaves tend to be higher in nitrogen and lower in secondary plant compounds than older leaves lower down the plant [45, 46]. This matches the behavior of this pest on greenhouse-grown pepper crops, in which young leaves on the upper section of the plant are the main target for feeding by larvae [47]. The height at which feeding took place broadly overlapped the height at which infected cadavers were found such that the probability of contact and necrophagy was much greater for infected compared to non-infected cadavers.

An additional aspect of baculovirus-induced climbing behavior in this insect was the observation that infected insects died close to the central stem of the plant. Such virus-mediated behavior could be adaptive as larvae moving over the plant often encountered the remains of the infected cadaver as they climbed up the plant stem and outwards to reach young leaves, thus increasing the likelihood of contact, necrophagy and virus transmission. Death at a site shaded by leaves in the uppermost 10% of the canopy and close to the central stem may also reduce exposure to solar ultraviolet radiation and increase the persistence of OBs on the plant surfaces [48, 49]. Death of infected insects close to the stem of the plant has not been reported previously as a characteristic of baculovirus-induced climbing behavior as far as we are aware, although unusually, nucleopolyhedrovirus-infected Winter moth larvae, *Operophtera brumata*, tended to move down the stem of host plants prior to death [50]. This resulted in contamination of host plant stems and transmission to early instar larvae that subsequently climbed up plant stems to feed on foliage. As such, given the high degree of host specificity of these viruses, baculovirus manipulation of insect behavior is likely to be adapted to the specific life cycle characteristics and feeding habits of each host insect species.

Differences were observed in the locations (vertical and horizontal distances) at which feeding, resting and walking behaviors were observed on pepper plants. As mentioned, feeding behavior is likely to be modulated by leaf chemistry, as larvae forage for leaves with high nutritional content and high digestibility [46]. In contrast, walking usually involved movement along leaf axils and up the plant’s central stem. Resting and walking behaviors were observed at similar locations. In some lepidopteran species larval displacement differs markedly between instars [51, 52], and this can affect the stage-specific probability of baculovirus infection when movement involves moving across a gradient of pathogen concentration on the host plant [53]. Displacement during foraging can also affect the probability of acquisition of a lethal dose of viral OBs, as infected larvae excrete large quantities of OBs in their feces or in gut regurgitate that can be transmitted to healthy conspecifics [11]. This was clear in our studies as a large fraction of the larvae that had no physical contact with infected cadavers themselves developed lethal polyhedrosis disease after moving over the plant on which an infected insect had foraged previously. This highlights the very high transmissibility of this virus [38].

Cannibalism and intraspecific necrophagy are effective mechanisms for the horizontal transmission of viruses in insect populations [54–56], including lepidopterans [22, 26, 27, 28, 57]. Interestingly, larvae of the Gypsy moth, *L*. *dispar*, were shown to be able to detect and avoid the remains of virus infected conspecifics [58]. These observations were confirmed by Parker et al. [59] who also demonstrated significantly reduced feeding behavior on foliage contaminated with viral OBs and heritable variation in the ability of larvae to detect and avoid the remains of infected cadavers. This contrasts with the findings of our study in which once an infected cadaver had been discovered, 33% (15/45) of *S*. *exigua* larvae consumed the infected cadaver in laboratory choice tests. Necrophagy on an infected cadaver was more frequently observed on pepper plants, with 73% (16/22) of larvae involved in this act. We did not examine the genetic basis or heritability of this behavior, so cannot determine whether larvae that avoided necrophagy were genetically distinct from necrophagous conspecifics. A preference for consumption of infected over non-infected cadavers was detected in *Helicoverpa armigera*, especially when several days had elapsed since the death of infected cadavers, suggesting that microbial decomposition may increase their attractiveness to necrophagous conspecifics [27].

Intraspecific necrophagy is clearly a risky behavior in insect populations that are infected by orally-transmitted virus pathogens. Necrophagy in *S*. *exigua* may therefore reflect specific nutritional requirements of larvae that develop on host plants in which certain nutrients, such as nitrogen, may be in short supply [60, 61]. In this respect, insect cadavers are likely to represent a rich source of proteins, fatty acids and other nutrients [62, 63]. Indeed, the prevalence of cannibalism in *Spodoptera* spp. increased when larvae developed on plants with low levels of nitrogen [64], or when the availability of alternative food sources was limited [26]. Similar results have been reported in other orders of insects, resulting in increased survival of necrophagous insects when conventional food resources were rare [65]. Moreover, some of the compounds produced during the decomposition of insect cadavers may be volatile and these can be used as cues by necrophagous insects to locate nutritional resources [66].

In conclusion, climbing in baculovirus infected insects has been shown to be a pathogen-induced behavior that increases the dispersal of viral OBs on the host plant as the insect cadaver disintegrates and OBs fall, or are washed by rainfall, over inferior plant foliage [3, 52, 67]. Despite greenhouse observations indicating that SeMNPV-infected cadavers were attractive to healthy conspecifics, laboratory choice tests and olfactometer studies provided no evidence for the existence of virus-associated olfactory or phagostimulant factors that might induce intraspecific necrophagy in *S*. *exigua* larvae. We conclude that baculovirus-induced climbing behavior, involving an increase in the height of infected larvae on the plant and their movement close to the central plant stem, increases the frequency of encounters between virus-infected cadavers and healthy larvae foraging for young foliage. This resulted in a very high incidence of intraspecific necrophagy; a behavior that invariably resulted in transmission of this lethal virus pathogen.

## Supporting Information

S1 Data Responses of experimental insects to infected and non-infected insect cadavers on spinach leaf disks in Petri dish areas (experiment 1). These results are summarized in the text and Fig 1.
(XLS)

S2 Data Results of choice test in which larvae were offered leaf disk sectors treated with (i) water vs. infected insect homogenate and (ii) infected insect homogenate vs. non-infected insect homogenate. Number of feeding events (for i and ii) and area consumed (for ii) are given. These results are shown in Fig 2A and 2B.
(XLS)

S3 Data Olfactometer response times (in seconds) for insects given a choice of infected vs. non-infected cadaver. The results of this study are described in the text for the corresponding olfactometer experiment.
(XLS)

S4 Data Results of insect behavior on plants with one infected and one non-infected cadaver. Hourly observations were performed on 22 individual healthy insects (replicates) over a 48 h period. At each observation insect activity was classified as feeding, walking or resting and the proximity of the experimental insect to the infected and non-infected cadavers was noted. The results are summarized in Fig 3A and 3B and Fig 4A and 4B.
(XLS)
